# Isolated Vaginal Agenesis Associated with Multiple Gastrointestinal Anomalies: A Case Report 

**DOI:** 10.21699/jns.v5i3.326

**Published:** 2016-07-03

**Authors:** R Angotti, F Molinaro, AL Bulotta, F Ferrara, M Sica, E Bindi, M Messina

**Affiliations:** Division of Pediatric Surgery, Department of Medical, Surgical and Neurological Sciences, University of Siena, Viale Bracci 53100 Siena, Italy

**Keywords:** Esophageal atresia, Anorectal malformation, Bowel duplication, Mullerian anomalies

## Abstract

More than 50% of infants with esophageal atresia have associated anomalies. We present a case report of a 46XX neonate with long-gap esophageal atresia and tracheoesophageal fistula (EA/TEF), anorectal malformation, bowel duplication and vaginal agenesis. This is an unusual association of abnormalities which had not yet described in literature.

## CASE REPORT

A female baby, weighing 2kg, was born by cesarean section at 36+5 gestational week. Antenatal ultrasound at 33rd gestational week showed polyhydraminos with absence of stomach bubble suspecting an esophageal atresia. A single umbilical artery was also identified. Apgar score at 1 and 5 minutes were very low 3 and 4, respectively. She was admitted to neonatal intensive care unit due to respiratory distress. She was intubated and mechanical ventilation was started. Nasogastric tube could not be passed. X-ray confirmed the esophageal atresia with tracheo-esophageal fistula. Physical examination showed an imperforate anus with recto-vestibular fistula (RVF). The external appearance of the genitalia seemed normal with swollen labia. The echocardiography excluded cardiac malformations. Abdomen ultrasound was normal. Karyotype was 46XX. The preoperative bronchoscopy showed slight tracheomalacia; Right transpleural thoracotomy and EA/TEF was repaired. During colostomy formation, ileal duplication, measuring 5cm in length and attached to antimesenteric border of proximal ileum, was found. It was resected and single-layer end to end ileo-ileal anastomosis performed. She was discharged in good clinical condition. At four months of age, the patient underwent posterior sagittal anorectoplasty (PSARP) for RVF. During operation, vaginal opening was not found. The colostomy was reversed 2 months after PSARP; during this operation internal genitalia showed a normal uterus, fallopian tubes, and ovaries. MRI performed also confirmed internal genitalia. At last follow up, she is 12 months old and doing fine. Vaginoplasty is planned at the prepubertal age after a psychological and gynecological evaluation.

## DISCUSSION

EA/TEF is frequently associated with other congenital malformations (36% to 66%)[1,2]. Cardiac malformations are the most common associated anomalies and their incidence is about 29%. Our patient had no cardiac anomaly. Incidence of pulmonary malformations is 6%. Tracheomalacia is associated in 11-33% patients of EA/TEF [3]. Our patient also had low grade tracheomalacia (type I). We are managing our patient conservatively as it has tendency to improve with passage of time [1,3]. 

Anorectal and genitourinary malformations are associated in 14% patients of EA/TEF [4]. RVF represents the most common type of anorectal defect seen in girls with anorectal malformation. Our patient had RVF, an ileal duplication cyst, and complete absence of vagina which was missed during initial evaluation of child at birth owing to swollen labia. It was identified during PSARP. The absence of vagina had psychological impact on the parents. We had to counsel them about further operation and fertility in future life of the child. Few cases of vaginal agenesis or distal vaginal atresia associated with anorectal malformations are reported in literature [4-6]. Our case can be considered unique because patient had anomalies of other systems such as EA/TEF, tracheomalacia, and ileal duplication cyst.

We have planned sigmoid colo-vaginoplasty at pre-pubertal age. The advantages of this bowel segment are related firstly to technical aspects, because isolation and mobilization are easy for its location and its blood supply, and secondly to its optimal features for neo vagina, because it's good size, length and spontaneous mucus production ensure an easy sexual activity.[7] We usually plan vaginal dilatations by Hegar dilator for at least 1 year after surgery which can be stopped if the patient begins sexual activity. 

In conclusion, in case of multiple anomalies in a patient like EA/TEF and anorectal malformations, every system should carefully be evaluated to identify other anomalies as delayed diagnosis may produce anxiety in parents if does not affect overall prognosis.

## Footnotes

**Source of Support:** Nil

**Conflict of Interest:** Nil
